# Intrarater and Interrater Reliability of Infrared Image Analysis of Forearm Acupoints before and after Moxibustion

**DOI:** 10.1155/2020/6328756

**Published:** 2020-09-26

**Authors:** Jiali Lou, Yongliang Jiang, Hantong Hu, Xiaoyu Li, Yajun Zhang, Jianqiao Fang

**Affiliations:** ^1^Department of Neurobiology and Acupuncture Research, The Third Clinical Medical College, Zhejiang Chinese Medical University, Key Laboratory of Acupuncture and Neurology of Zhejiang Province, Hangzhou 310053, China; ^2^The Third Affiliated Hospital of Zhejiang Chinese Medical University, Hangzhou City, Zhejiang Province, China

## Abstract

The objective of this study was to determine the intrarater and interrater reliabilities of infrared image analysis of forearm acupoints before and after moxibustion. In this work, infrared images of acupoints in the forearm of 20 volunteers (M/F, 10/10) were collected prior to and after moxibustion by infrared thermography (IRT). Two trained raters performed the analysis of infrared images in two different periods at a one-week interval. The intraclass correlation coefficient (ICC) was calculated to determine the intrarater and interrater reliabilities. With regard to the intrarater reliability, ICC values were between 0.758 and 0.994 (substantial to excellent). For the interrater reliability, ICC values ranged from 0.707 to 0.964 (moderate to excellent). Given that the intrarater and interrater reliability levels show excellent concordance, IRT could be a reliable tool to monitor the temperature change of forearm acupoints induced by moxibustion.

## 1. Introduction

In recent years, in light of technological advances, various kinds of modern techniques have been applied widely in the research on the modernization of traditional Chinese medicine. Among them, multiple techniques are applied to investigate the biological properties of acupoints and meridians, such as laser Doppler [1, 2], functional near infrared spectroscopy [3, 4], and infrared thermography (IRT) [5, 6]. Given that IRT can measure mid to longwave infrared radiation emanating from all objects and convert it into a temperature value [7], it can analyze the distribution of skin surface temperature sensitively and efficiently, and previous studies indicated that IRT is an aid tool to investigate the thermal properties of acupoints. Moreover, it could monitor the temperature change of acupoints induced by stimulation of acupuncture or moxibustion. For instance, Yang et al. [8] applied IRT to capture facial temperature changes before and after moxibustion at Zusanli (ST 36) and Hegu (LI 4), the results of which revealed that moxibustion at different acupoints could evoke specific facial temperature changes. Deng et al. [9] applied laser acupuncture to stimulate Houxi (SI 3) of the small intestine meridian, and they observed a significant temperature increase of 1.5°C in the distal site.

Nevertheless, it is worth noting that potential subjectivity and inaccuracy might exist in such studies when IRT raters analyze the temperature values of acupuncture points in thermographic images through analysis software. For instance, Valente et al. [10] analyzed the intrarater and interrater reliabilities of infrared image analysis of facial acupoints in patients with facial paralysis, the results of which indicated that the reliability in temperature analysis of acupoints was influenced by the incomplete consistency in determining acupoint positions when repeatedly positioning the same acupoint manually. That is to say, the reliability in acupoint temperature tends to have close correlations with the accurate positioning of corresponding acupoints in thermographic images by IRT raters. However, the anatomical signs of acupoints, which can aid the precise positioning of acupoints, are not very distinct in thermographic images; thus, the IRT raters have to locate acupoints based on individual experience in such circumstances. In addition, due to the large amount of temperature data underlying a great number of thermographic images, it is a common phenomenon that multiple IRT raters are involved in the temperature analysis of acupoints. As a result, these internal and external confounding factors may increase artiﬁcial error, thereby reducing the credibility of the temperature analysis of acupoints. Therefore, it is of great significance to determine the intrarater and interrater reliabilities of the temperature analysis of acupoints.

Based on the meridian theory, there are many acupoints with special functions distributed in the forearm, which are often used in clinical practice and scientific research [11]. Therefore, the present study selected forearm acupoints as regions of interest (ROIs) to determine the intrarater and interrater reliabilities of infrared image analysis before and after moxibustion.

## 2. Materials and Methods

### 2.1. Subjects

Twenty healthy volunteers were recruited from the Third Affiliated Hospital of Zhejiang Chinese Medical University, including 10 men and 10 women (age, 29.05 ± 3.73 years). Healthy subjects were included based on a recent physical examination report, which could confirm they had no major systemic diseases, such as cardiovascular, respiratory, digestive, urinary, hematological, endocrine, and neurological diseases [12]. Exclusion criteria were as follows: (1) subjects were taking medication; (2) subjects had a fever in the last seven days; (3) subjects had visible skin damage or scars in the forearm; and (4) subjects in pregnancy or lactation.

All subjects were fully informed of the study protocol and informed consent forms were signed. This study was approved by Ethics Committee of the Third Affiliated Hospital of Zhejiang Chinese Medical (approval number: ZSLL-KY-2019-001G-01).

### 2.2. Experimental Design and Moxibustion Procedure

In this study, suspended moxibustion was applied to evoke temperature change in ROIs in the left forearm for infrared image analysis, which was performed by holding an ignited moxa stick at an appropriate distance above the subject's skin surface.

**Figure 1:**
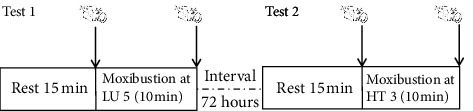
Measurement procedure.

The experimental designs consisted of two tests. In test 1, moxibustion was performed at the acupoint LU 5 of the lung meridian for 10 mins in all subjects, during which two thermographic images of the forearm was taken (one before moxibustion and the other one immediately after 10 min moxibustion). In test 2, the acupoint HT 3 of the heart meridian was stimulated in the same way in all subjects. Two tests were performed at an interval of 72 hours to avoid interexperiment interference. The schematic of the experiment is shown in Figure 1.

### 2.3. Acquisition of IRT Images

All IRT examinations were carried out in a quiet room with temperature controlled at 26 ± 1°C and relative humidity controlled at 50%–60%. There were no direct sunshine and no abnormal radiant source in the room. Prior to formal IRT examinations, all subjects were requested not to drink any alcoholic or caffeine-containing products and avoid violent exercise within 24 hours. All subjects were stabilized for 30 minutes in a bed in the room before IRT examinations. They were also informed to keep silent and avoid limb movement during the whole measuring period.

A thermograph camera (NEC InfRec R450, Avio Infrared Technologies Co., Ltd., Tokyo), with a measurement range from −20 to 60°C and a resolution of 640 × 480 pixels, was used to obtain thermographic images. By adjusting the height and angle of the camera, the left forearm of the subjects was displayed distinctly in the camera screen. Two thermographic images were collected before and immediately after 10 min moxibustion.

### 2.4. Temperature Analysis of ROIs

**Figure 2:**
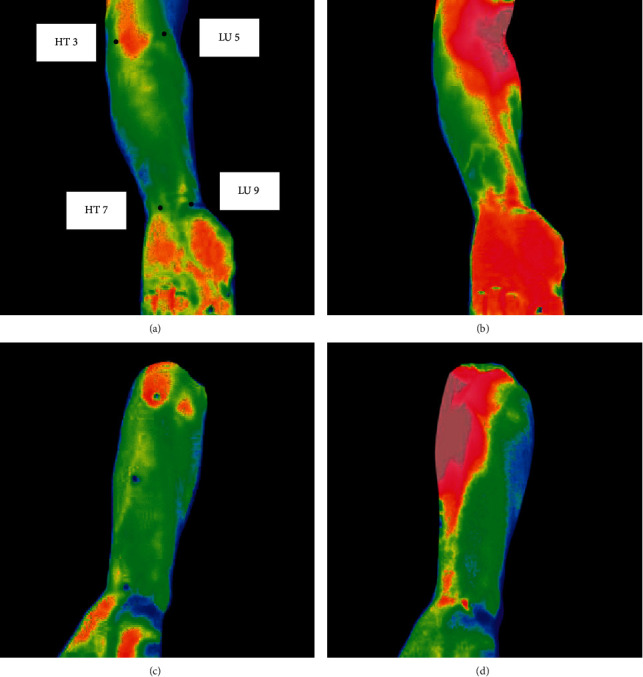
Distribution of the 4 ROIs in the thermographic images of the forearm before and after moxibustion. (a) Before moxibustion at LU 5; (b) after moxibustion at LU 5 for 10 min; (c) before moxibustion at HT 3; and (d) after moxibustion at HT 3 for 10 min.

All acquired thermographic images were imported into the analysis software NS9500Std (Avio Infrared Technologies Co., Ltd., Tokyo); 4 ROIs in the left forearm were analyzed, which corresponded to the following 4 forearm acupoints (shown in Figure 2): (1) Taiyuan (LU 9); (2) Chize (LU 5); (3) Shenmen (HT 7); and (4) Shaohai (HT 3). Anatomical locations of these 4 forearm acupoints for IRT analysis are displayed in Table 1.

In this study, the parameter for the ROI was set up as a standard circular region with a diameter of 1 cm (about 61 pixels), which was equal to the size of an acupoint [13]. The average temperature value of ROI (*T*_ROI_) was used to represent the temperature of each acupoint. In addition, IRT raters calculated the temperature difference (Δ*T*) of each ROI before and after 10 min moxibustion. In order to analyze the intrarater and interrater reliabilities, two trained IRT raters performed the temperature analysis of acupoints based on thermographic images (40 groups in total and 2 thermal images in each group) in two different periods at a one-week interval.

### 2.5. Statistical Analysis

The statistical analysis was performed using the SPSS software, version 24.0 (Chicago, USA). Temperature data were calculated using mean and standard deviation (SD). Change in temperature before and after moxibustion were calculated using mean and standard error (SE). The intrarater and interrater reliabilities was determined based on the ICC, with its respective 95% confidence interval. Interpretation of ICC values was based on that proposed by Fleiss [14]. Levels of reliability coefficients were classiﬁed as follows: excellent (more than 0.90), substantial (0.75–0.90), moderate (0.40–0.75), and low (less than 0.40). In addition, Bland and Altman plots were used to intuitively verify the degree of agreement.

## 3. Results

The results of repeated IRT measurements for 20 subjects are shown in Table 2. As illustrated, the mean temperature range of forearm acupoints was from 31.56 to 32.22°C.

As shown in Tables 3 and 4, the Δ*T* values of the lung meridian (LU 9) were higher than the heart meridian (HT 3 and HT 7) in test 1, during which moxibustion was performed at the acupoint LU 5 of the lung meridian. Meanwhile, the Δ*T* values of the heart meridian (HT 7) were higher than the lung meridian (LU 9 and LU 5) in test 2, during which moxibustion was applied at the acupoint HT 3 of the heart meridian.

Table 5 illustrates the ICC values of intrarater and interrater reliabilities of forearm acupoint temperature measurement before moxibustion. ICC values of intrarater ranged from 0.766 to 0.994, and the lowest value appeared at LU 5, with a substantial reliability level. Furthermore, ICC values of interrater ranged from 0.707 to 0.944, and the lowest value occurred at LU 5, with a moderate reliability level.

ICC values of intrarater and interrater in the acupoint Δ*T* measurement after moxibustion are shown in Tables 6 and 7. ICC values of intrarater ranged from 0.758 to 0.987, and the lowest value appeared at LU 5, with a substantial reliability level. Besides, ICC values of interrater ranged from 0.754 to 0.968, and the lowest value occurred at HT 3, with a substantial reliability level.

**Figure 3:**
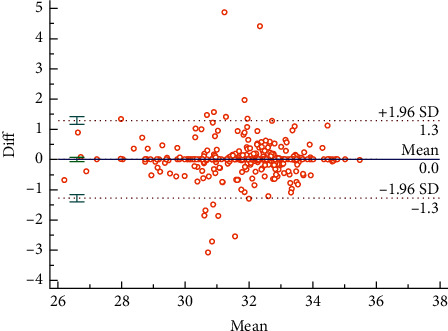
Bland–Altman plot for intrarater agreement between two sets of the temperature values, 7.18% of all readings taken fell outside the 95% agreement limits.

**Figure 4:**
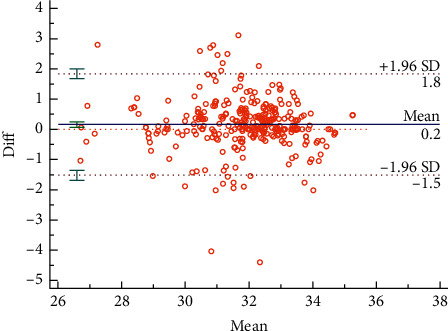
Bland–Altman plot for interrater agreement between two sets of the temperature values, 6.87% of all readings taken fell outside the 95% agreement limits.

**Figure 5:**
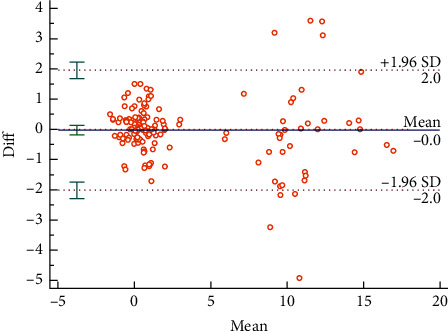
Bland–Altman plot for intrarater agreement between two sets of temperature differences (Δ*T*) after moxibustion at LU 5, 5.00% of all readings taken fell outside the 95% agreement limits.

**Figure 6:**
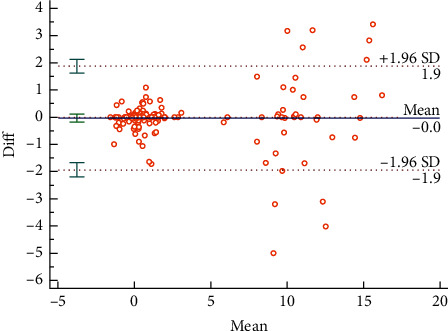
Bland–Altman plot for interrater agreement between two sets of temperature differences (Δ*T*) after moxibustion at LU 5, 6.25% of all readings taken fell outside the 95% agreement limits.

**Figure 7:**
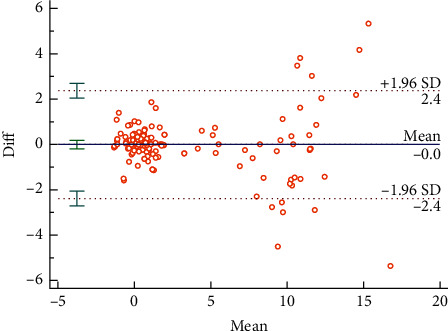
Bland–Altman plot for intrarater agreement between two sets of temperature differences (Δ*T*) after moxibustion at HT 3, 6.87% of all readings taken fell outside the 95% agreement limits.

**Figure 8:**
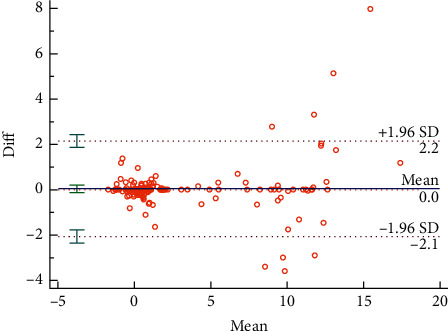
Bland–Altman plot for interrater agreement between two sets of temperature differences (Δ*T*) after moxibustion at HT 3, 5.00% of all readings taken fell outside the 95% agreement limits.

The Bland–Altman plots were used to visualize the consistency between the two sets of measurements. Figures 3 and 4 reveal the consistency of intrarater and interrater before moxibustion, among which 7.18% and 6.87% of all data distributed outside the 95% agreement limits, respectively. Figures 5–8 show the consistency of intrarater and interrater after moxibustion, among which 5.00% and 6.87% of all data fell outside the 95% agreement limits.

## 4. Discussion

In recent years, a great number of studies have adopted IRT as an aid tool to investigate the thermal properties of acupoints or dynamically monitor the temperature change of acupoints induced by acupuncture, moxibustion, or massage [15–17]. However, few studies have verified the reliability of acupoint temperature analysis, thereby reducing the validity and robustness of corresponding research results. One study found that there were different reliability values in analyses of IRT in different acupoint sites, indicating that the reliability of acupoint temperature analyses had close correlations with the precise positioning of acupoints in thermographic images by IRT raters [10]. As far as we are concerned, this is the ﬁrst study aiming to determine the intrarater and interrater reliability of infrared image analysis of forearm acupoint before and after moxibustion.

Intrarater and interrater reliabilities refer to the consistency of different measurements, which was determined by ICC values. In the present study, the results show moderate to strong reproducibility in two assessors within the two sessions at a one-week interval. Moreover, the Bland–Altman plots of our study data further veriﬁed the consistency of results. As shown in Figures 3–6, almost all of the data points were within the range of 95% CI, thereby indicating that the consistency of our study data was validated.

Based on preliminary experiments, we found that moxibustion could evoke more significant changes in the infrared images of acupoints at the distal end of the meridian than other acupuncture modalities, such as electroacupuncture or manual acupuncture. Thus, we chose moxibustion as the stimulation modality. As a result, we observed the temperature significantly rise in the distal acupoint, the distance of which was far away from the local moxibustion acupoint. It is worth noting that, regarding the effect of moxibustion on the skin temperature in the distal sites along the stimulated meridian, results of different studies were conflicting. For instance, Plakornkul et al. [18] selected Laogong (PC 8) of the pericardium meridian as the moxibustion point, and they found that the temperature of PC 8 increased, accompanied by the temperature decrease in middle fingertips. In contrast, another study found that moxibustion on Mingmen (GV 4) could induce a high-temperature path along the Governor Vessel (GV) and raise skin temperature in distal areas of the GV [19]. In the present study, regardless of moxibustion at the heart meridian or the lung meridian, the temperature in the distal site along the stimulated meridian tended to rise.

Some studies indicated that the circulation path of meridians seems to be characterized by better thermal conductivity [20, 21]. Meridians can be sensitized by moxibustion on local acupoints, during which microcirculation becomes more exuberant, oxygen partial pressure of the deep tissue rises, and energy metabolism turns more vigorous along the circulation route of meridians [22]. This may explain that why the temperature of the distal acupoint of the stimulated meridian, which was far away from the local moxibustion acupoint, could rise significantly. Furthermore, previous studies indicate that meridians are anatomically associated with nerves, blood vessels, and lymphatic vessels [23, 24]. These anatomical structures are generally distributed in the longitudinal direction in the forearm. This might explain that moxibustion-induced temperature change of the acupoints (HT 3 in test 1 or LU 5 in test 2) that located lateral to the moxibustion site was nonsignificant, although they were located very close to the moxibustion site. Nevertheless, the present study mainly focused on the intrarater and interrater reliabilities of infrared image analysis of forearm acupoints before and after moxibustion, the specificity between different meridians, and acupoints induced by moxibustion will be further elaborated in our future research.

This study had some limitations to be addressed. First, the study population was limited to healthy young adults, and the thermal response may be different for other age groups or unhealthy populations. Second, the sample size was small, which might lead to a low statistical power.

## 5. Conclusions

In conclusions, the temperature measurements of forearm acupoints before and after moxibustion yielded excellent intrarater and interrater reliabilities. Thus, IRT can be a reliable tool to investigate thermal properties of acupoints and dynamically monitor the temperature change of forearm acupoints induced by acupuncture or moxibustion. Nevertheless, current evidence might not robust against the inclusion criteria of specific participants and small sample size, and more research studies are needed to verify current findings.

## Figures and Tables

**Table 1:** Anatomical locations of 4 forearm acupoints for IRT analysis.

Forearm acupoints	Anatomical locations
Taiyuan (LU 9)	On the anterolateral aspect of the wrist, between the radial styloid process and the scaphoid bone, in the depression ulnar to the abductor pollicis longus tendon.
Chize (LU 5)	On the anterior aspect of the elbow, at the cubital crease, in the depression lateral to the biceps brachii tendon.
Shenmen (HT 7)	On the anteromedial aspect of the wrist, radial to the flexor carpi ulnaris tendon, on the palmar wrist crease.
Shaohai (HT 3)	On the anteromedial aspect of the elbow, just anterior to the medial epicondyle of the humerus, at the same level as the cubital crease.

**Table 2:** Mean and standard deviation results of acupoint temperature values collected by two different raters in two sessions before moxibustion.

Acupoint	Rater 1, *T*_ROI_ (mean ± SD)		Rater 2, *T*_ROI_ (mean ± SD)	
	Session 1	Session 2	Session 1	Session 2
LU 9	31.98 ± 1.63	31.84 ± 1.66	31.86 ± 1.69	31.85 ± 1.63
LU 5	31.83 ± 1.04	31.83 ± 1.11	31.56 ± 1.01	31.62 ± 1.05
HT 7	32.22 ± 1.85	32.10 ± 2.06	32.01 ± 2.13	32.01 ± 2.01
HT 3	31.80 ± 1.40	31.89 ± 1.32	31.62 ± 1.44	31.69 ± 1.38

**Table 3:** Results of acupoint temperature difference (Δ*T*) of each ROI after moxibustion at LU 5.

Acupoint	Rater 1, Δ*T*(mean ± SE)		Rater 2, Δ*T* (mean ± SE)	
	Session 1	Session 2	Session 1	Session 2
LU 9	0.88 ± 0.22	0.87 ± 0.22	0.90 ± 0.23	0.99 ± 0.24
LU 5	10.77 ± 0.65^*∗*^	10.83 ± 0.61^*∗*^	11.02 ± 0.66^*∗*^	10.98 ± 0.47^*∗*^
HT 7	−0.04 ± 0.20	0.17 ± 0.22	−0.08 ± 0.21	−0.07 ± 0.17^*∗*^
HT 3	0.37 ± 0.12	0.33 ± 0.11	0.35 ± 0.14	0.33 ± 0.13

Note: *∗P* < 0.05, compared with Δ*T* of LU 9.

**Table 4:** Results of acupoint temperature difference (Δ*T*) of each ROI after moxibustion at HT 3.

Acupoint	Rater 1, Δ*T*(mean ± SD)		Rater 2, Δ*T* (mean ± SD)	
	Session 1	Session 2	Session 1	Session 2
LU 9	0.34 ± 0.16	0.34 ± 0.21	0.27 ± 0.22	0.33 ± 0.21
LU 5	0.36 ± 0.15	0.36 ± 0.18	0.28 ± 0.16	0.30 ± 0.15
HT 7	0.97 ± 0.26	0.95 ± 0.29	0.87 ± 0.29	0.92 ± 0.28
HT 3	10.12 ± 0.73^*∗*^	10.03 ± 0.65^*∗*^	10.45 ± 0.72^*∗*^	10.11 ± 0.46^*∗*^

Note: *∗P* < 0.05, compared with Δ*T* of HT 7.

**Table 5:** Intrarater and interrater reliabilities of temperature measurement of forearm acupoints before moxibustion.

Acupoint	Intrarater reliability				Interrater reliability			
	Rater 1		Rater 2		Session 1		Session 2	
	ICC	Scale	ICC	Scale	ICC	Scale	ICC	Scale
LU 9	0.863	Substantial	0.994	Excellent	0.873	Substantial	0.797	Substantial
LU 5	0.808	Substantial	0.766	Substantial	0.831	Substantial	0.707	Moderate
HT 7	0.904	Excellent	0.984	Excellent	0.891	Substantial	0.853	Substantial
HT 3	0.885	Substantial	0.913	Excellent	0.832	Substantial	0.944	Excellent

**Table 6:** Intrarater and interrater reliabilities of temperature difference measurement of forearm acupoints after moxibustion at LU 5.

Acupoint	Intrarater reliability				Interrater reliability			
	Rater 1		Rater 2		Session 1		Session 2	
	ICC	Scale	ICC	Scale	ICC	Scale	ICC	Scale
LU 9	0.922	Excellent	0.961	Excellent	0.816	Substantial	0.864	Substantial
LU 5	0.934	Excellent	0.758	Substantial	0.892	Substantial	0.758	Substantial
HT 7	0.948	Excellent	0.947	Excellent	0.848	Substantial	0.819	Substantial
HT 3	0.969	Excellent	0.987	Excellent	0.870	Substantial	0.872	Substantial

**Table 7:** Intrarater and interrater reliabilities of temperature difference measurement of forearm acupoints after moxibustion at HT 3.

Acupoint	Intrarater reliability				Interrater reliability			
	Rater 1		Rater 2		Session 1		Session 2	
	ICC	Scale	ICC	Scale	ICC	Scale	ICC	Scale
LU 9	0.881	Substantial	0.881	Substantial	0.889	Substantial	0.836	Substantial
LU 5	0.934	Excellent	0.934	Excellent	0.943	Excellent	0.868	Substantial
HT 7	0.978	Excellent	0.978	Excellent	0.947	Excellent	0.964	Excellent
HT 3	0.904	Excellent	0.904	Excellent	0.850	Substantial	0.754	Substantial

## Data Availability

The data used to support this study are made available from the corresponding author upon request.

## Abbreviation

IRT: Infrared thermography
ICC: Intraclass correlation coefficient
ROI: Regions of interest
SD: Standard deviation
SE: Standard error
*T*_ROI_: Temperature value of ROI
Δ*T*: Temperature difference
GV: Governor Vessel.
